# Identification of Apple Fruit-Skin Constitutive Laws by Full-Field Methods Using Uniaxial Tensile Loading

**DOI:** 10.3390/ma17030700

**Published:** 2024-02-01

**Authors:** Teresa Campos, Rafael Araújo, José Xavier, Quyền Nguyễn, Nuno Dourado, José Morais, Fábio Pereira

**Affiliations:** 1 CMEMS-UMINHO, Universidade do Minho, 4800-058 Guimarães, Portugalnunodourado@dem.uminho.pt (N.D.); 2 LABBELS–Associate Laboratory, 4800-058 Guimarães, Portugal; 3 CITAB/UTAD, Departamento de Engenharias, Quinta de Prados, 5001-801 Vila Real, Portugaljmorais@utad.pt (J.M.); famp@utad.pt (F.P.); 4 UNIDEMI, Department of Mechanical and Industrial Engineering, NOVA School of Science and Technology, Universidade NOVA de Lisboa, 2829-516 Caparica, Portugal; 5 LASI, Intelligent Systems Associate Laboratory, 4800-058 Guimarães, Portugal; 6 2C2T-Centro de Ciência e Tecnologia Têxtil, Universidade do Minho, 4800-058 Guimarães, Portugal

**Keywords:** apple skin, hyperelasticity, digital image correlation, uniaxial tensile loading, finite element analysis

## Abstract

The protective and preservative role of apple skin in maintaining the integrity of the fruit is well-known, with its mechanical behaviour playing a pivotal role in determining fruit storage capacity. This study employs a combination of experimental and numerical methodologies, specifically utilising the digital image correlation (DIC) technique. A specially devised inverse strategy is applied to evaluate the mechanical behaviour of apple skin under uniaxial tensile loading. Three apple cultivars were tested in this work: Malus domestica Starking Delicious, Malus pumila Rennet, and Malus domestica Golden Delicious. Stress–strain curves were reconstructed, revealing distinct variations in the mechanical responses among these cultivars. Yeoh’s hyperelastic model was fitted to the experimental data to identify the coefficients capable of reproducing the non-linear deformation. The results suggest that apple skin varies significantly in composition and structure among the tested cultivars, as evidenced by differences in elastic properties and non-linear behaviour. These differences can significantly affect how fruit is handled, stored, and transported. Thus, the insights resulting from this research enable the development of mathematical models based on the mechanical behaviour of apple tissue, constituting important data for improvements in the economics of the agri-food industry.

## 1. Introduction

Skin appearance and cracking are significant causes of fruit value decrease and losses. It is well recognised that fruit skin (FS; exocarp) is subjected to a complex stress field during fruit growth, harvesting, storage and transportation. Additionally, the complex mechanical behaviour of FS makes the identification of its constitutive laws a challenging theme, presenting difficulties from both theoretical and experimental perspectives. Understanding the relationship between FS composition and morphology and its mechanical behaviour is an important research topic that has a clear economic impact on the agri-food industry.

The majority of existing works on this issue involve the evaluation of material parameters of fruit tissues (e.g., rupture force, rupture energy and firmness), aiming to establish relationships between mechanical properties and production and post-harvesting factors. Oey et al. [1] highlighted the influence of turgor on the structural and mechanical properties of apple tissue. Also, Juxia, W. and co-authors [2] studied the biomechanical characteristics of the peels of two apple cultivars, using tensile strength, tear strength and puncture resistance tests. The Allende team [3] focused on evaluating the relationship between the histology of tomato peel and its breaking strength. Grotte M. and his co-workers [4] measured the firmness of the skin and flesh of fruit using puncture tests.

In the above studies, the experimental and data-reduction methods used do not allow for the identification of intrinsic mechanical properties of FS, which are fundamental to numerical studies when estimating fruit mechanical behaviour.

The quality and risk of rupture of FS are usually evaluated by parameters such as rupture force, rupture energy, and firmness. These parameters are often associated with high scatter, primarily due to the mechanical tests as well as the coarse geometric and mechanical simplifications adopted in evaluating these parameters. The classical method used to determine the mechanical properties of FS is the uniaxial tensile test [5,6]. Different loading strategies have been used for strain partition into elastic, plastic, and viscoelastic components [7,8,9].

It is known that plant tissues have complex mechanical behaviour [10] and most of them are considered to be elastic or viscoelastic [7,8,9]. However, some reported values of ultimate strains [5,10] suggest that a hyperelastic approach is required, although the material behaviour can change from elastic (viscoelastic) to hyperelastic (hyperviscoelastic) through growth and ripening phases. Bargel and Neinhuis [5] confirm this behaviour, showing the decrease in extensibility of the tomato fruit cuticle at the final stages of ripening. Similarly, Bidhendi et al. [11] noted that stress–strain behaviour of the onion epidermis under tension was remarkably non-linear. These authors evaluated the capacity of a few hyperelastic models to reproduce the non-linear deformation of the onion epidermis using a fitting strategy. Over the years, an extensive amount of hyperelastic constitutive models have been proposed, such as the generalised Fung model [12], the Yeoh model [13,14], and the Holzapfel–Gasser–Ogden model [15,16]. In this context, it remains unclear which specific experimental tests should be conducted to precisely calibrate a hyperelastic model.

In this work, we investigated the hyperelastic behaviour of apple skin coupled with full-field displacement measurements based on the digital image correlation technique. Monotonic tensile tests were performed to obtain stress–strain curves on apple skin specimens in the longitudinal direction. A methodology based on inverse identification considering finite element model updating (FEMU) was adopted to minimise the difference between full-field numerical and experimental displacements. Through this process, the Yeoh model coefficients were determined to replicate the hyperelastic response of the skin accurately. This approach allows for the rational exploration of the connections between mechanical properties, material composition, and morphology.

## 2. Materials and Methods

### 2.1. Specimen Preparation

**Figure 1:**
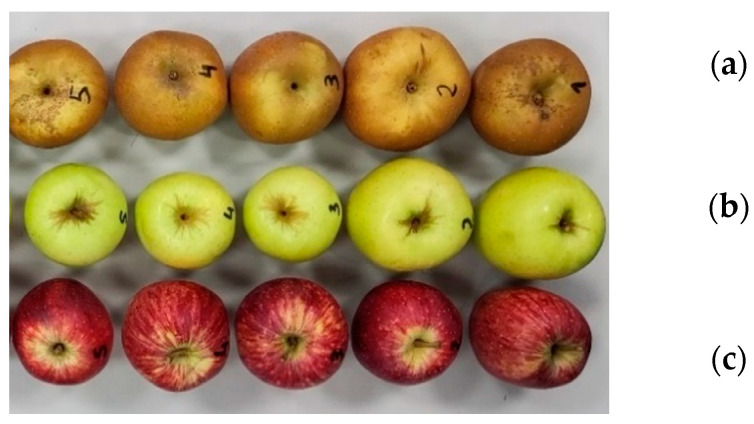
Apple cultivars: (**a**), Malus pumila Rennet; (**b**) Malus domestica Golden Delicious; and (**c**) Malus domestica Starking Delicious.

Three apple cultivars, Malus domestica Starking Delicious, Malus pumila Rennet, and Malus domestica Golden Delicious (Figure 1), were tested under the same conditions (optimal stage), one day after harvesting in the Armamar region (Portugal; 41°07′ N 7°41′ O). Five skin samples were extracted from each cultivar from the epicarp region, where lenticel zones were less concentrated. Skin samples were taken from trees with no shading area, without any control over the sunning position.

**Figure 2:**
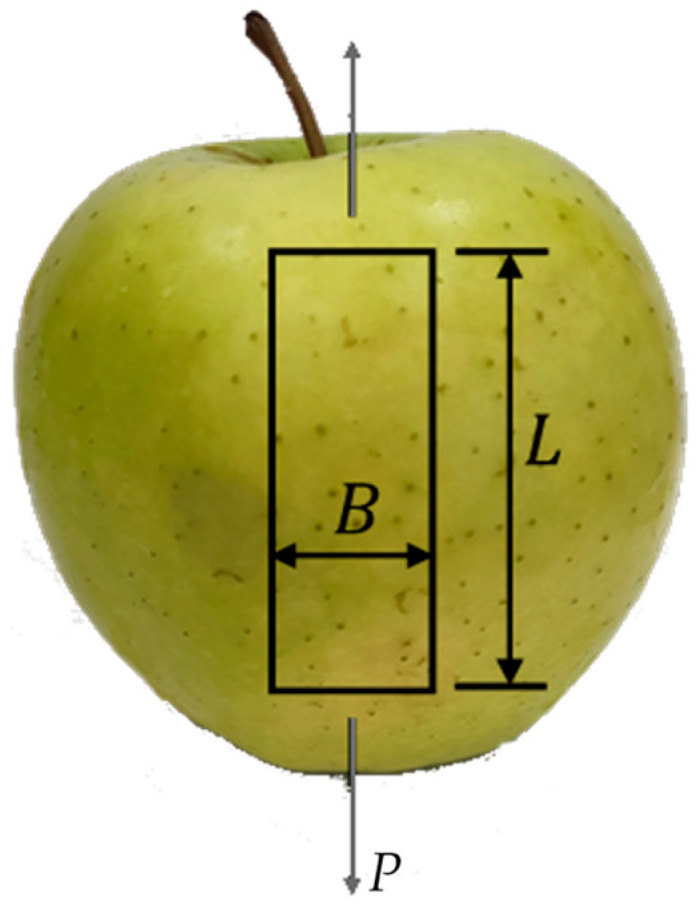
Specimen orientation (L=60 mm ,  B=20 mm).

This was performed using a scalpel to form a rectangle shape (60×20 mm) in the longitudinal direction (Figure 2). Adequate razoring was then executed to remove apple pulp until a uniform thickness (0.2 mm) was attained along the entire length of the specimen. Subsequently, a speckle pattern suitable for DIC measurements was applied to the pill side (20×20 mm) by spraying black paint using an airbrush over the natural substrate of the apple skin (Figure 2).

### 2.2. Uniaxial Tensile Test

**Figure 3:**
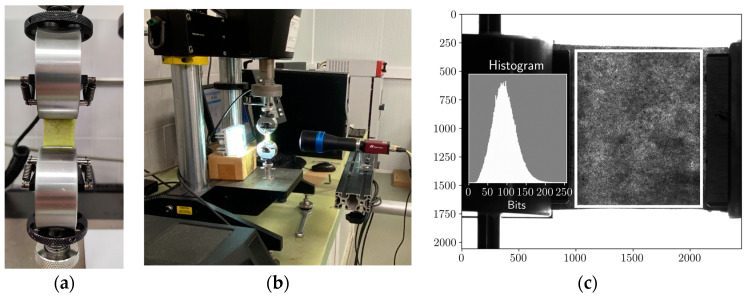
(**a**) Detail of the uniaxial tensile test; (**b**) Experimental setup showing DIC acquisition system; (**c**) DIC pattern with the corresponding histogram.

Tensile tests were performed in the apple skin specimens (in the longitudinal direction: pedicel to calix) using a servo-electrical testing machine (Micro-Tester INSTRON 5848; 2 kN load-cell, INSTRON, MA, USA), with the crosshead displacement rate set to 3 mm/min (Figure 3a). To prevent water loss, the test was completed immediately after the specimen preparation, at room temperature (25 °C and 65 RH). Specimen slipping in the grips was avoided by cautious grip tightening during the specimen setting. Load–displacement curves were monitored by setting the acquisition rate to 5 Hz while capturing images for DIC (digital image correlation) measurements (Figure 3b) with a frequency of 1 Hz. These time steps allowed for the synchronisation of the load data with the acquired DIC images. Light distribution was adequately chosen to allow suitable contrast for DIC measurements (Figure 3c).

### 2.3. Digital Image Correlation

#### 2.3.1. Optical System and Speckle Pattern

The integration of the 2D-DIC technique with mechanical tests was chosen due to its suitability for conducting contactless and full-field measurements across the fruit-skin substrate. This approach eliminates the need for gluing-based systems, such as strain gauges or Bragg gratings, traditionally used to assess strains [17,18,19]. An 8-bit camera (Baumer Optronic GmbH, Radeberg, Germany, model FWX20) with a telecentric lens (Opto Engineering SRL, Mantova, Italy, model TC 13 36) was used, as reported in Table 1. Images were recorded at an acquisition frequency of 1 Hz, and the working distance was set to 103.5 mm, with a fixed magnification factor of 4.4 µm/pixel. This camera sensor was positioned perpendicular to the flat surface of sample while ensuring there was appropriate lighting with a white-light LED (Table 1). The MatchID DIC software was used to process the DIC analysis.

#### 2.3.2. DIC Setting: Parametric Analysis

When conducting DIC measurements, it is essential to perform a convergence analysis on the intrinsic parameters that govern the numerical imaging correlation method [17,20].

This is particularly crucial for biological materials that exhibit natural heterogeneities at the observation scale. The DIC parameters significantly impact spatial resolution and accuracy in both displacement and strain measurements [21]. A parametric study was conducted with the MatchID Performance Analysis Tool [22], allowing for multiple DIC analyses on the same image set and considering various parameter combinations. Each point on the DIC setting space corresponds to a specific spatial resolution. To quantify this metric, we used the Virtual Strain Gauge (VSG) measure [22,23]:

VSG = [(SW − 1) × ST] + SS [pixels] (1)

where SW refers to the strain window, determining the number of data points in the fitting polynomial approach; ST stands for the subset step; and SS represents the subset size.

The physical units of millimetres for the VSG can be achieved by considering the image conversion factor of the optical system (see Table 1). Each parameter was systematically examined within a defined design space with specified minimum and maximum values (refer to Table 2). We assessed the reconstruction of the strain component $\varepsilon_{xx}$ at the specimen centre, observing its variation in response to the preselected DIC setting parameters.

**Figure 4:**
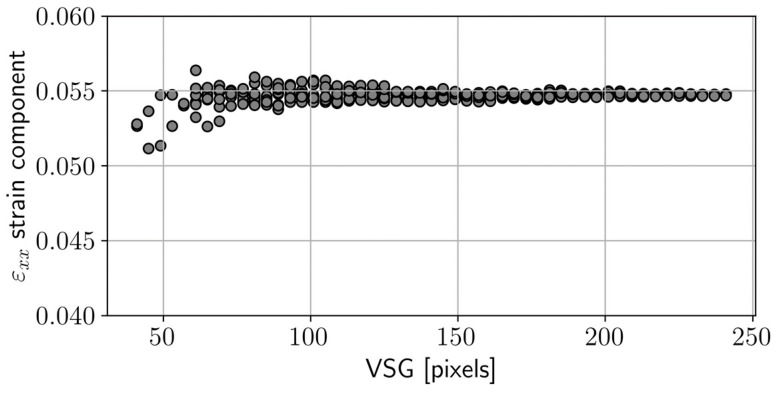
Evaluation of $\varepsilon_{xx}$ as a function of the coordinate *x* for several Virtual Strain Gauge (VVSG) values for both tension and compression tests.

Figure 4 shows the $\varepsilon_{xx}$ strain signal at the central reference point as a function of the VSG. The data points visibly converge to a plateau with increasing VSG values, suggesting an average strain at that specific point. This analysis revealed a compromise that balanced spatial resolution and accuracy. Finally, Table 3 summarises the DIC settings selected in this study to report the full-field data.

### 2.4. Procedure to Assess the Material Law

Evolutionary algorithms (EAs) have proven to be effective when applied to the iterative optimisation of FEMU updating. This iterative process involves defining an objective function based on the differences between the nodal displacements in experimental and numerical datasets. Noteworthy applications encompass tasks such as determining viscoelastic properties for wood-based panels [24], identifying elastic parameters at fibre–matrix interfaces in composite materials [25], optimising parameters for hyperelastic cardiac materials [26], and establishing damage parameters for large-scale structural systems [27]. Furthermore, EAs offer the advantage of not being heavily reliant on the initial solution, thus mitigating the risk of becoming trapped in local minima during the optimisation process [24,28].

The Yoeh, Odgen, and Neo-Hookean hyperelastic models are the most recommended hyperelastic models in the literature for simulating the mechanical behaviour of various biological tissues. MCalibration 5.1.2 software (Veryst Engineering, Needham, MA, USA) was used to identify which of the three suggested models could most accurately replicate the experimental outcome (i.e., stress–strain curves). Experimental stress–strain (σ=f(ε)) data ensuing from the tensile tests was used as input to identify the material law. The objective function to be minimised was set as the mean square difference (MSD) between the numerical output and the experimental data (σ=f(ε)). An extensive search for optimal parameters minimising the fitness function was performed, which included an initial random search, followed by the application of an EA. This search strategy (i.e., EA) was offered by MCalibration as a suitable methodology to identify the hyperelastic coefficients, thus leading to the most suitable agreement with the experimental true σ−ε curve. The accuracy of the obtained solutions was heavily contingent upon the selected evolutionary parameters, such as the population size, crossover rate, and mutation rate (Table 4). In this process, Yeoh’s model demonstrated more suitability in replicating the experimental response.

**Figure 5:**
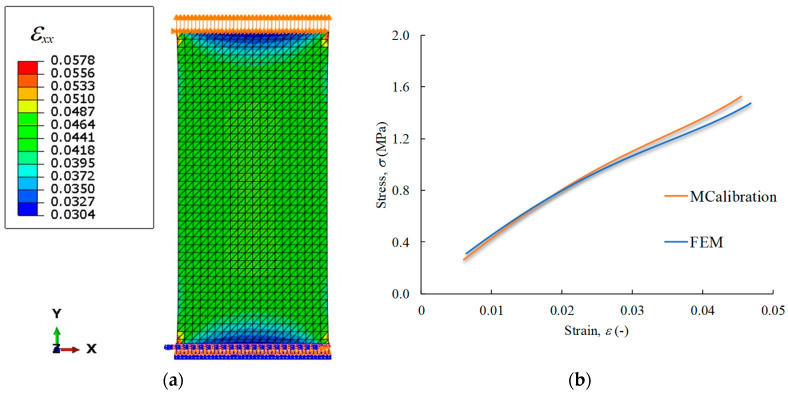
Numerical results: (**a**) FE mesh showing the strain field along the loading direction; and (**b**) the attained stress–strain agreement of FE and MCalibration.

The output of this procedure is the identification of the set of coefficients defining the material law, allowing us to replicate the experimental (mechanical) behaviour under tensile loading. To this aim, in-plane stress analyses were conducted considering a finite element model formed by 3321 shell (6-node) quadrilateral finite elements, using boundary conditions to ensure admissible kinematic requirements for the tensile test, as illustrated in Figure 5a.

## 3. Hyperelastic Law

The approach adopted in this work starts with the basic assumption that apple skin exhibits an isotropic behaviour. Concerning the hyperelastic models currently used in commercial FEM software, the deformation gradient F and the right Cauchy–Green tensor C are decomposed into dilatational and distortional components,

(2) $$F=J{\;}^{1/3}\;F^{*}$$

(3) $$C=F^{T}F=J{\;}^{2/3}C^{*}$$

with $F^{*}$ and $C^{*}$ being the distortional components and J=det(F) being the dilatational component. Accordingly, the first and second invariants of $C^{*}$ are related to the invariants of the right Cauchy–Green tensor by the equations:

(4) $$I_{1}^{*}=J^{-\frac{2}{3}}I_{1}\;\;\mathrm{and}\;\;I_{2}^{*}=J^{-\frac{4}{3}}I_{2}$$

$I_{1}$, $I_{2}$ and $J^{2}$ are the invariants of the right Cauchy–Green tensor

(5) $$I_{1}=\lambda_{1}^{2}+\lambda_{2}^{2}+\lambda_{3}^{2}\;I_{2}=\lambda_{1}^{2}\lambda_{2}^{2}+\lambda_{2}^{2}\lambda_{3}^{2}+\lambda_{3}^{2}\lambda_{1}^{2}\;I_{3}=\lambda_{1}^{2}\lambda_{2}^{2}\lambda_{3}^{2}=J^{2}$$

and $\lambda_{1}$, $\lambda_{2}$ and $\lambda_{3}$ are the principal extension ratios.

The general constitutive equation of hyperelastic materials provdes the Cauchy stress tensor σ as a function of the deformation gradient F by

(6) $$\sigma \left(F\right)=\frac{1}{J}\frac{\partial \psi \left(F\right)}{\partial F}F^{T}$$

$\psi \left(F\right)$ is the strain-energy density function. The restrictions imposed by the principle of material frame indifference imply that the strain-energy density function depends only on the stretch tensor (U) component of F or, equivalently, on $C=U^{2}$:

(7) $$\psi \left(F\right)=\hat{\psi }\left(C\right)$$

The chain rule yields the following relationship between the partial derivatives of the two strain-energy density functions with respect to their tensor arguments:

(8) $$\frac{\partial \psi \left(F\right)}{\partial F}=2F\frac{\partial \hat{\psi }\left(C\right)}{\partial C}$$

Hence, combining Equations (6) and (8), the Cauchy stress can be expressed as

(9) $$\sigma \left(C\right)=\frac{2}{J}F\frac{\partial \hat{\psi }\left(C\right)}{\partial C}F^{T}$$

For isotropic materials, the strain-energy density function is a function of the right Cauchy–Green tensor via its invariants (4 and 5), and can be written as $\tilde{\psi }\left(I_{1}^{*}\left(I_{1},J\right),I_{2}^{*}\left(I_{2},J\right),J\right)$. Thus, the Cauchy stress tensor (9) becomes:

(10) $$\sigma \left(I_{1}^{*},I_{2}^{*},J\right)=\frac{J}{2}\left(\frac{\partial \tilde{\psi }}{\partial I_{1}^{*}}\frac{\partial I_{1}^{*}}{\partial I_{1}}+J^{2/3}I_{1}^{*}\frac{\partial \tilde{\psi }}{\partial I_{2}^{*}}\frac{\partial I_{2}^{*}}{\partial I_{2}}\right)b-\frac{2}{J}\frac{\partial \tilde{\psi }}{\partial I_{2}^{*}}\frac{\partial I_{2}^{*}}{\partial I_{2}}b^{2}+\left(\frac{\partial \tilde{\psi }}{\partial I_{1}^{*}}\frac{\partial I_{1}^{*}}{\partial J}+\frac{\partial \tilde{\psi }}{\partial I_{2}^{*}}\frac{\partial I_{2}^{*}}{\partial J}+\frac{\partial \tilde{\psi }}{\partial J}\right)I$$

where $b=FF^{T}$ is the left Cauchy–Green tensor and I is the identity tensor.

(11) $$\hat{\psi }\left(C_{ij};D_{i}\right)=\overset{N}{\underset{i+j=1}{\sum }}C_{ij}{\left(I_{1}^{*}-3\right)}^{i}{\left(I_{2}^{*}-3\right)}^{j}+\overset{N}{\underset{i=1}{\sum }}\frac{1}{D_{i}}{\left(J-1\right)}^{2i}$$

where $C_{ij}$ and $D_{i}$ represent material parameters. A particular version of the polynomial model is the Yeoh model:

(12) $$\hat{\psi }\left(C_{10},C_{20},C_{30},\kappa \right)=C_{10}\left(I_{1}^{*}-3\right)+C_{20}{\left(I_{1}^{*}-3\right)}^{2}+C_{30}{\left(I_{1}^{*}-3\right)}^{3}+\frac{\kappa }{2}{\left(J-1\right)}^{2}$$

with κ representing the bulk modulus.

## 4. Results and Discussion

### 4.1. Uniaxial Tensile Tests

**Figure 6:**
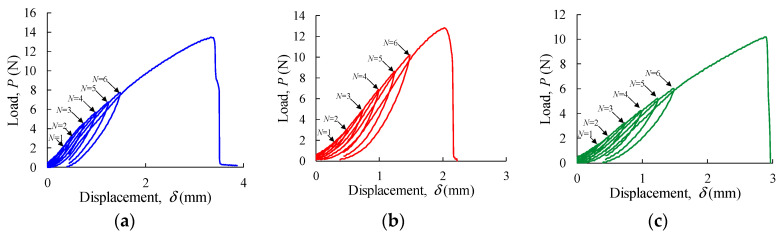
Load–unloading cycles in: (**a**) Malus domestica Starking Delicious; (**b**) Malus pumila Rennet; and (**c**) Malus domestica Golden Delicious.

Figure 6 shows the load–displacement (*P*-*δ*) curves obtained cyclically, with increasing load levels at a constant crosshead displacement rate (0.5 mm/min) until the final rupture (last cycle). These results show that the area enclosed by the loading–unloading curves, representing the energy per unit volume, gradually increases with the load value defined to invert the crosshead, thus configuring a hysteresis loop (a history-dependent property). This shows the increase in width in the hysteresis loop strain with load amplitude and the rise in compliance with the number of load cycles in the loading branch, with the latter effect being referred to as softening behaviour. Another characteristic identified in those plots regards the unrecoverable strain in the material following unloading, which is more visible as the load value defined to invert the crosshead increases. Figure 6 also illustrates that the material can no longer replicate the same non-linear behaviour, as the number of cycles increases, as observed from N=1 to N=6. As a result, it is obvious that the load–unload curves obtained from mechanical testing on apple skin exhibit non-linear elastic behaviour. This response suggests that following pure elasticity, these materials can tolerate a high intensity of load and even exceed 100% deformation without damage [29].

**Figure 7:**
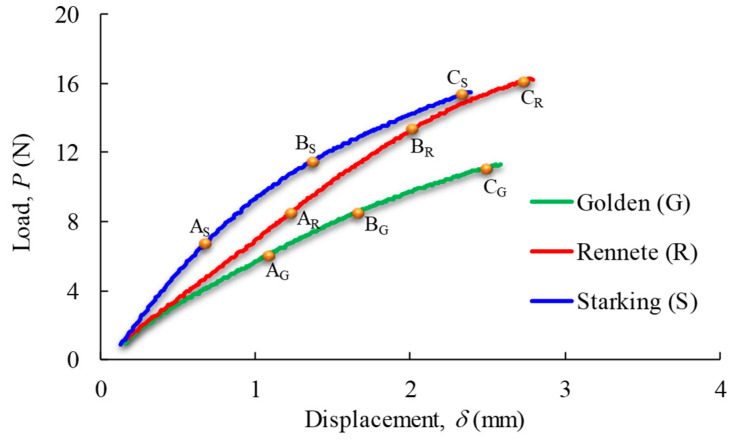
Typical load–displacement curves under tensile loading. Points A, B, and C identify the following stages: within the elastic response, the occurrence of relevant strain gradients (lenticels identification), and the crack onset of apple skin, respectively.

Figure 7 shows the set of experimental P-δ curves obtained in monotonic tensile tests for the tested apple cultivars. These results evidence the existence of a linear trend in the load–displacement curve (up to point A), followed by non-linear behaviour until total failure.

**Figure 8:**
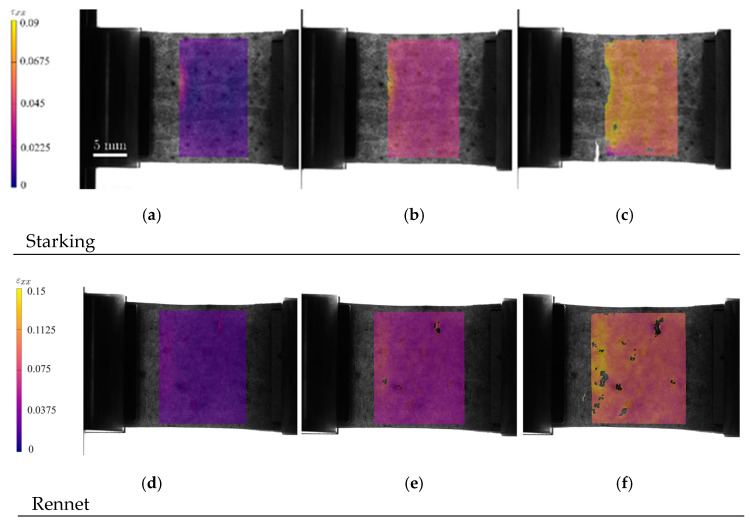

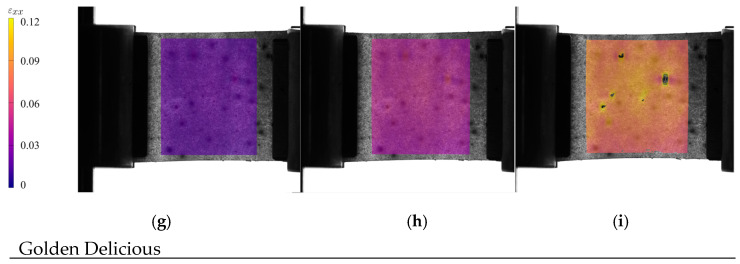
Strain cartographies of Starking Delicious, Rennet, and Golden Delicious in the x direction for longitudinal loading ($\varepsilon_{xx}$), obtained in three load phases according to Figure 7 in points: (**a**) A_S_; (**b**) B_S_; (**c**) C_S_; (**d**) A_R_; (**e**) B_R_; (**f**) C_R_; (**g**) A_G_; (**h**) B_G_; and (**i**) C_G_.

**Figure 9:**
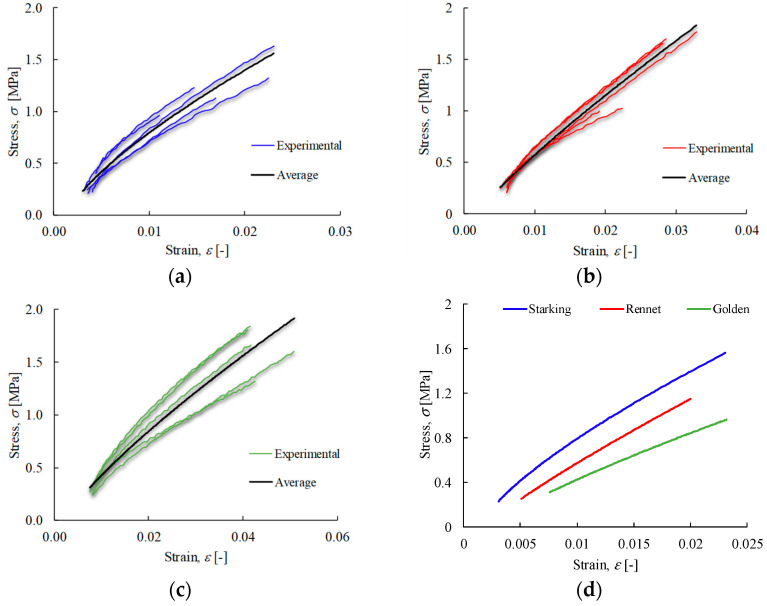
True stress–strain curves of apple cultivars: (**a**) Malus domestica Starking Delicious; (**b**) Malus pumila Rennet; (**c**) Malus domestica Golden Delicious; and (**d**) corresponding average curves.

DIC processing has revealed a set of strain cartographies along the loading axis, i.e., $\varepsilon_{xx}$ (Figure 8a,d,g), where an extensive homogeneous region was formed in the specimen central area. Mean values of strain $\varepsilon_{xx}$ were calculated considering the measurements performed in that region, which were correlated with the applied load to plot the stress–strain curves for each apple cultivar (Figure 9a–c). Those results allowed us to conclude the existence of a low scatter of the ensued experimental data. Figure 9a–c also presents the result of regressions derived from fitting procedures using power-law distributions for each cultivar, showing clear differences.

**Figure 10:**
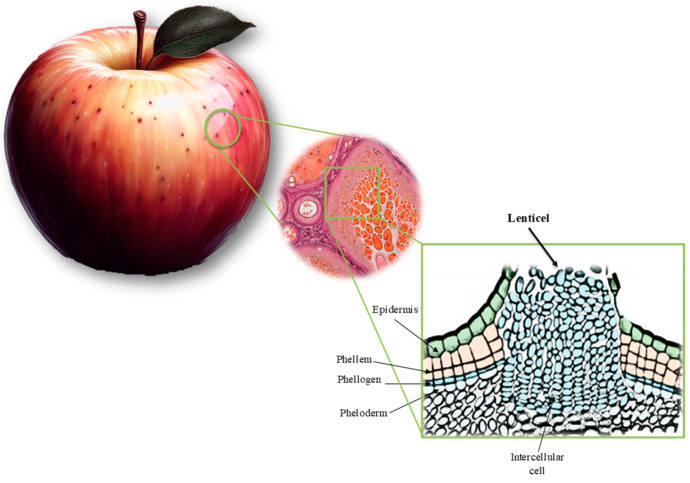
Lenticels schematic illustration.

Figure 8b,e,h allow us to identify strain gradients (points B in Figure 7) in particular regions of the skin apple samples, whose origin was later associated with existing anatomic structures in apple skin, namely lenticels (Figure 10). The existence of these structures in the apple skin allows for ensuring fruit oxygen and other gas exchanges, which are characteristic of the secondary plant body (rather than the stomata, or pores in the epidermis, in the primary plant). In fact, lenticels appear as a result of microcracks in the bark cuticle, similar to the formation of areas known as russet. While lenticels form small spots on the skin, russet covers large areas of the fruit. Both phenomena involve periderm formation in response to rupture in the cuticle, influencing skin mechanical behaviour [30].

In regards to Figure 8c,f,i, with correspondence to point C in Figure 7, crack initiation and propagation are visible in the set of regions previously identified as highly affected by strain concentrations (Figure 8b,e,h), where damage onset was under development.

### 4.2. Numerical Agreement

**Figure 11:**
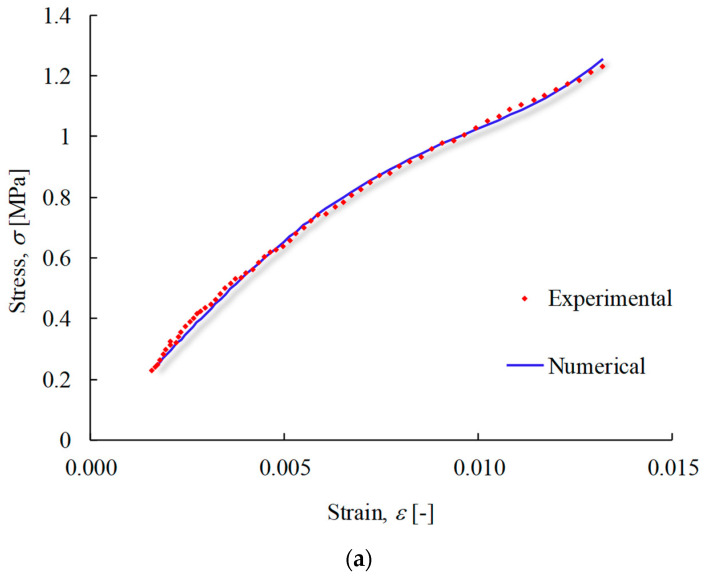

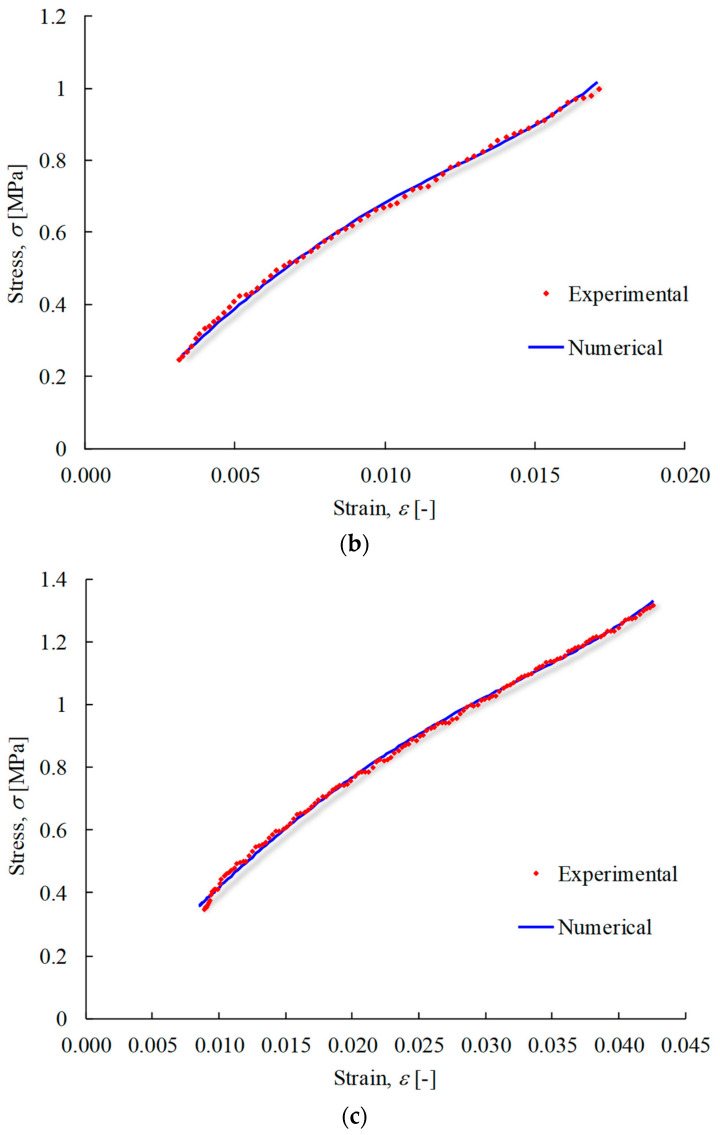
True stress–strain curve showing the obtained numerical agreement: (**a**) Malus domestica Starking Delicious; (**b**) Malus pumila Rennet; and (**c**) Malus domestica Golden Delicious.

A procedure was developed to identify the most suitable constitutive model to mimic the hyperelastic behaviour of apple skin under uniaxial tensile loading. The methodology was based on the stress–strain response, exhibiting a pronounced non-linear S-shape (as noticed by [2]). Implementing this approach, the Yeoh model proved very effective in replicating the true stress–strain response, encompassing the non-linear domain (see Figure 11; rescaled to improve visibility). This observation suggests that the adopted methodology is suitable for extrapolating situations beyond experimentally tested conditions, including scenarios involving different strain rates or environmental conditions.

Table 5 presents the set of coefficients used to define the constitutive law characteristic of the Yeoh model (Equation (12)) for each cultivar identified by the inverse method.

The results presented in Table 6 reveal a significant scatter in the identified coefficients regardless of the apple cultivars. Despite this, coefficient *C*_10_ presents a smaller CoV in comparison to *C*_20_ and *C*_30_, demonstrating a more consistent response to smaller strains (as noted by Yeoh (1990) [13]), both within the individual apple cultivar and among cultivars.

**Figure 12:**
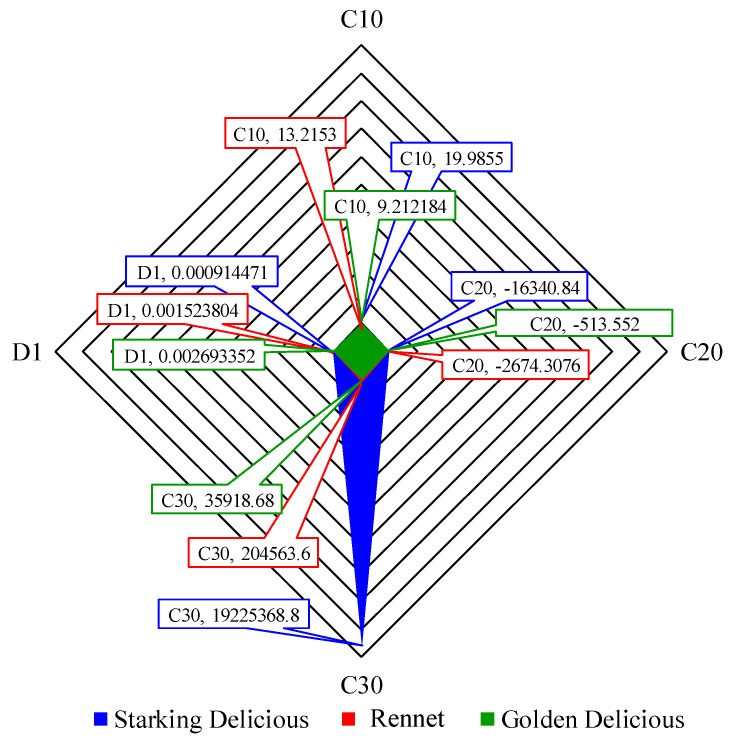
Average of three cultivars.

Also, mean values of coefficients *C*_10_, *C*_20_ and *C*_30_ were determined to perceive eventual differences among the analysed cultivars (Figure 12). Analysing C_20_ and C_30_ for Starking, Rennet and Golden, the difference among these values is very significant, which evidences existing potential intrinsic differences in the apple skin microstructure or composition. Although a negative *C*_20_ may appear unconventional, one can affirm that there are no real physical issues with this observation [13,14]. The fact that coefficient *C*_20_ is negative, whereas *C*_10_ and *C*_30_ are positive, indicates that the secant shear modulus varies with the deformation in a characteristic way [13,14]. Fruit skin was considered isotropic and nearly incompressible. For the numerical analysis, compressibility values close to 0 were assigned to *D*_1_ (in MPa).

The coefficients for Yeoh’s hyperelastic model on the studied apple cultivars are compared in Table 6 with other research on various biological materials. A broad range of values in various biological materials was seen when compared with literature studies, underscoring the uniqueness of the coefficients for each kind of tissue. In comparison to apple skin, the epidermis of onions has much lower values, suggesting a more elastic response. These biological materials have specific mechanical properties that underscore the need to tailor constitutive models to capture their particularities. This underscores the requirement for a customised approach when examining various biological structures.

## 5. Conclusions

This work follows an innovative approach that harnesses a combination of experimental techniques based on full-field measurements (contactless) with appropriate numerical modelling to predict the mechanical response of apple skin under tensile loading.

The proposed methodology is used to identify the coefficients of a constitutive law of apple skin, to mimic the hyperelastic behaviour. In this process, the Yeoh’s model appeared to be the most appropriate. The influence of the lenticel areas on the mechanical behaviour of the apple skin was emphasised by DIC measurements. According to this study, these areas are at risk of rupturing and are important in lowering the apple skin’s resistance to external stress before achieving irreversible deformation. The identification of the influence of lenticel areas on the mechanical behaviour of apple skin adds an additional layer of practical relevance.

Moreover, modelling and predicting the mechanical behaviour of these materials was rendered attainable through the biomechanical interpretation of both Yeoh’s model parameters and deformation cartographies. Understanding the coefficients in Yeoh’s equation makes it possible to develop more accurate models to explain the mechanical response of apple skin, which enhances the comprehension of the non-linear and elastic skin characteristics. In the future, this mechanical characterisation may allow for establishing a relationship between constitutive parameters of fruit skin and qualitative attributes currently used to classify fruit (e.g., firmness and texture).

These findings constitute an important contribution to our knowledge of the mechanical behaviour of this complex biological structure (apple skin), outlining practical implications concerning the selection and application of apple cultivars in many technical and commercial contexts, from the food industry to biomaterials engineering. Additionally, these results not only allow for advances in the scientific understanding of apple skin behaviour but also have valuable practical implications in sectors that depend on the quality and durability of this fruit.

## Figures and Tables

**Table 1:** Optical devices and DIC data.

**CCD Camera**	Model	Baumer^®^ Optronic FWX20 (8 bts)
	Pixel resolution	1624 × 1236 pixels, 4.4 µm/pixel
	Shutter time	0.7 ms
	Acquisition frequency	1 Hz
**Lens**	Model	Opto Engineering Telecentric lens TC 23 36
	Magnification	0.243 ± 3%
	Field of view (1/1.8″)	29.3 × 22.1 mm^2^
	Working distance	103.5 ± 3 mm
	Working F-number	f/8
	Field depth	11 mm
	Conversation Factor	0.018 mm/pixel
**Lighting**	LED	Raylux 25
**Speckle Pattern**	Painting technique	Airbrush (nozzle set of 0.2 mm)
	Average speckle size	6 pixels|21.5 µm

**Table 2:** Parameters used in the Performance Analysis Tool of MatchID 2D DIC.

**Subset-based settings**	
Subset size	SS ∈ {21 + 4*n* | *n* =10} pixel
Subset step	ST = 10 pixel (fixed)
Shape function	{Affine, Quadratic}
**Strain reconstruction-based settings**	
Strain window	SW ∈ {3 + 2*n | n* = 8} data points
Polynomial order ^⋆^	Bilinear (Q4), Biquadratic (Q8)
Strain convention	Green–Lagrange

^⋆^ Local least-squares fitting approach for the strain evaluation.

**Table 3:** DIC and strain settings selected in the full-field measurements.

**DIC settings**	
Correlation criterion	ZNCC
Interpolant	Bicubic spline
Subset shape function	Quadratic
Subset size	41 pixels
Step size	10 pixels
Image pre-filtering	Gaussian, 5-pixel kernel
**Strain settings**	
Strain window size	Nine data points
Strain interpolation	Quadratic Q4
Strain convention	Green–Lagrange

**Table 4:** Parameters used in the evolutionary search.

Population Size	Crossover Rate	Mutation Rate	Maximum Number of Generations
100	0.05	0.95	1000

**Table 5:** Material parameters of the Yeoh model identified by the inverse method for each apple cultivar (values *C*_10_, *C*_20_, *C*_30_ in MPa).

Starking Delicious							
Material parameters	Specimen 1	Specimen 2	Specimen 3	Specimen 4	Specimen 5	Average	CoV (%)
*C*_10_	13.28	30.85	24.37	17.74	13.68	19.99	34%
*C*_20_	−2289.75	−56,083.60	−17,502.80	−3771.46	−2056.59	−16,340.8	−127%
*C*_30_	509,610	82,163,500	11,920,400	960,077	573,257	19,225,369	165%
Rennet Delicious							
Material parameters	Specimen 6	Specimen 7	Specimen 8	Specimen 9	Specimen10	Average	CoV (%)
*C*_10_	11.69	13.61	13.53	13.56	13.68	13.22	6%
*C*_20_	−908.19	−1672.44	−4534.94	−1476.87	−4779.09	−2674.31	−61%
*C*_30_	123,028	311,363	137,444	252,213	198,770	204,563.6	34%
Golden Delicious							
Material parameters	Specimen 11	Specimen 12	Specimen 13	Specimen 14	Specimen 15	Average	CoV (%)
*C*_10_	7.354	10.33	10.30	7.187	10.88	9.21	17%
*C*_20_	−304.38	−503.81	−762.77	−384.85	−611.95	−513.55	−32%
*C*_30_	16,769	33,889.7	65,864.2	23,801.7	39,268.8	35,918.68	47%

**Table 6:** Yeoh’s coefficients of different biological tissues.

Material	Yeoh Coefficients		
	C10 (MPa)	C20 (MPa)	C30 (MPa)
Starking Delicious (This study)	19.99	−16,340.8	19,225,369
Rennet (This study)	13.22	−2674.31	204,563.6
Golden Delicious (This study)	9.21	−513.55	35,918.68
Onion epidermis [11]	0.406	6.68	34
Porcine skin [31]	0.26	15.5	1.75
Pig skin [32]	8.42	8.75	2.35
Arteries branches [33]	0.1067	5.1602	0.0

## Data Availability

Data are contained within the article.
